# The impact of internet-based cognitive behavioral therapy on mental health outcomes and life in breast cancer patients: a systematic review and meta-analysis

**DOI:** 10.3389/fonc.2024.1434581

**Published:** 2024-10-08

**Authors:** Jianlong Han, Yunxin Ding, Hongwei Wang, Qing Li, Huanqie Zhai, Shuling He

**Affiliations:** ^1^ Jiamusi University School of Clinical Medicine, Jiamusi University, Jiamusi, China; ^2^ Department of General Surgery, The First Affiliated Hospital of Jiamusi University, Jiamusi, China; ^3^ Tianjin Mental Health Center, Tianjin, China; ^4^ Department of Ophthalmology, The Fourth Affiliated Hospital of Harbin Medical University, Harbin, China

**Keywords:** internet-based cognitive behavioral therapy (ICBT), breast cancer, mental health outcomes, life, systematic review, meta-analysis

## Abstract

**Background:**

Internet-based cognitive behavioral therapy(ICBT) improves the impact of breast cancer through online platforms, modular learning, goal setting, relaxation exercises, and other techniques. Compared to traditional cognitive behavioral therapy (CBT), ICBT offers advantages such as the convenience of flexible time and location choices and reduced manpower requirements. In recent years, research exploring the impact of ICBT on breast cancer patients has been increasing, with conflicting results across different studies. Therefore, the purpose of this study was to comprehensively examine the impact of ICBT on the psychological health and quality of life of breast cancer patients through a systematic review and meta-analysis.

**Methods:**

We searched ten databases in both English and Chinese, with the search period extending from the inception of the databases to December 30, 2023. Literature screening, bias risk assessment, data extraction, and evidence level evaluation were independently conducted by two researchers. All the data were analyzed using RevMan 5.4 and Stata 17.0 software.

**Results:**

A total of 2079 breast cancer patients were included in this study, of which 1171 patients received ICBT treatment. The results show that ICBT can reduce anxiety [SMD=-0.19, 95%CI (-0.37, -0.01), P=0.0008] and depression [SMD=-0.20, 95%CI (-0.37, -0.02), P=0.001], alleviate fatigue [SMD=-0.34, 95%CI (-0.67, -0.01), P=0.04], and improve quality of life [SMD=0.20, 95% CI (0.03, 0.38), P=0.02] in breast cancer patients. However, the intervention effects of ICBT on insomnia [SMD=-0.44, 95%CI (-0.93, 0.06), P=0.08] and sleep quality [SMD=-0.14, 95%CI (-0.30, 0.01), P=0.06] in breast cancer patients are not significant. The subgroup analysis showed that when the intervention period is longer than 8 weeks, the number of intervention modules exceeds 6, and a waitlist control group is included, there is a significant effect on reducing patients’ anxiety and depression. However, the method of guidance and whether the intervention period exceeds 12 weeks are not related.

**Conclusion:**

ICBT can alleviate anxiety and depression in breast cancer patients, with the intervention effects being independent of the guidance method. Significant results were obtained when the intervention period was >8 weeks and the number of modules was >6. ICBT can reduce fatigue and improve quality of life in breast cancer patients, but its impact on sleep quality was not significant. More high-quality research is needed in the future.

**Systematic review registration:**

PROSPERO, identifier CRD42024494744.

## Introduction

In 2020, the incidence of breast cancer surpassed that of lung cancer, and breast cancer became the most common cancer (1). With advancements in disease screening and treatment methods, the survival rate of breast cancer patients has been continually increasing. However, surgical treatment and its associated complications may lead to body image disturbances, causing patients to feel a sense of physical incompleteness and experience a decrease in self-esteem (2, 3). Moreover, patients are also concerned about cancer recurrence (4), enduring significant psychological distress, and are at higher risk of developing anxiety and depression than the general population, which can have an adverse impact on patients’ mental health and reduce their treatment adherence (5), exacerbate symptoms such as pain (6), and severely affect their prognosis (6). Therefore, it is essential to explore effective treatment methods to improve the impact of breast cancer, thereby facilitating their recovery and enhancing their quality of life.

CBT facilitates mental health and emotional regulation by modifying patients’ ways of thinking and behavioral patterns. It enhances breast cancer patients’ understanding of their condition, subsequently improving their psychological state and life (6), and assists them in actively confronting the disease and solving problems (7). Currently, CBT has been proven by numerous studies to effectively alleviate negative emotions and improve patients’ quality of life, outperforming other therapies. Numerous studies have demonstrated that CBT can effectively reduce patients’ depression and anxiety and improve their quality of life, and is superior to other treatment methods (8–12). Traditional CBT can be divided into individual and group formats and is conducted through face-to-face interventions by therapists. However, the limited number of therapists cannot meet the needs of all breast cancer patients (13), and traditional CBT is typically conducted at fixed times and locations, which can easily conflict with various aspects of a patient’s daily life. Additionally, its implementation may be constrained by geographical factors and limited access to local healthcare resources, making it difficult to widely implement in clinical practice (14). With the advancement of information technology and the internet’s penetration into various aspects of life, interventions provided online offer better operability (15). ICBT is not limited by time and space, allowing patients to choose their treatment time based on personal circumstances (16), better protecting patient privacy and enhancing treatment compliance.

Given this, it remains unclear whether ICBT can alleviate psychological distress in breast cancer patients and improve the impact of breast cancer. Additionally, the impact of different guidance methods, intervention frequency, and intervention duration on the effectiveness of the intervention is also uncertain. This study aimed to evaluate the impact of ICBT on the negative emotions and quality of life of breast cancer patients, providing a basis for the application of ICBT in this patient population. Relevant meta-analysis results show that ICBT can alleviate anxiety and depression in cancer patients (20, 21). Their study included patients with all types of cancer, lacking specific analysis focused on breast cancer patients. Therefore, the effectiveness of ICBT in breast cancer patients remains uncertain. Currently, an increasing number of studies are exploring the effects of ICBT on breast cancer patients, but the results vary across studies. Therefore, this research aims to evaluate the impact of ICBT on the psychological health outcomes and quality of life of breast cancer patients by analyzing whether factors such as different guidance methods, intervention durations, and the number of modules influence these outcomes. The findings will provide evidence for the application of ICBT in breast cancer patients.

## Methods

### Study design

This study was conducted according to the Preferred Reporting Items for Systematic Reviews and Meta-Analyses guidelines [PRISMA 2020 checklist (**Appendix S1**)] (19). Moreover, this study has been registered on the international prospective register of systematic reviews (PROSPERO) with the registration number CRD42024494744.

### Literature search

We searched four Chinese databases (China Biology Medicine disc (CBM), China National Knowledge Infrastructure (CNKI), VIP Database, and Wanfang Database) and six English databases (Embase, Web of Science, PubMed, Cochrane Library, CINAHL, APA PsycInfo). The search period was from the establishment of the database to December 30, 2023. To ensure comprehensive coverage, we limited the search criteria to intervention methods and target populations. The search strategy was composed of the following search terms: ‘internet’, ‘online’, ‘web’, ‘net’, ‘mobile’, ‘cognitive behavioral therapy’, ‘ICBT’, ‘eCBT’, ‘cCBT’, ‘wCBT’, ‘TCBT’, ‘breast tumor*’, ‘breast cancer’, and ‘mammary tumor*’. We did not conduct a search for grey literature. References of the included literature were screened to incorporate previous relevant studies (**Appendix S3**).

### Study selection

We adhered to the PICOS criteria to establish our inclusion criteria, which included the following: (1) study type: randomized controlled trials; (2) population: patients clinically diagnosed with breast cancer, aged ≥18 years, regardless of occupation or race; (3) interventions and comparisons: the experimental group received ICBT, whereas the control group received standard care, placebo interventions, or were put on a waitlist; (4) Outcomes: anxiety, depression, fatigue, sleep, and quality of life. The exclusion criteria were as follows: (1) the experimental group combined with other interventions; (2) duplicate publications or those whose full texts were inaccessible; (3) reviews, conference abstracts, etc. Two researchers used EndNote X9 software to conduct a preliminary screening by reading titles and abstracts, followed by a second screening through reading the full text of the literature. After the screening was completed, the two researchers cross-checked, and if there was disagreement, a discussion was held. If the result could not be determined after discussion, a third researcher made the decision.

### Data extraction

Two researchers independently extracted the following information from the included literature using a predesigned information sheet: (1) basic information of the literature: author, year of publication, country, sample size, and age of subjects; (2) intervention content: guidance methods, number of intervention modules, content of intervention, duration of single intervention, frequency of intervention, intervention period, and control conditions; (3) measurement tools and outcomes of the outcome indicators.

### Risk of bias and grading of evidence

Two researchers independently assessed the risk of bias in the included studies using the evaluation handbook recommended by the Cochrane Collaboration (22), covering seven aspects. In the case of disagreements, the two researchers discussed to reach a decision, if a consensus could not be reached, a third researcher made the final decision. Furthermore, we applied the grading of recommendations assessment, development and evaluation (GRADE) approach to categorize the level of evidence for each outcome into four levels: very low, low, moderate, and high (22). The quality assessment was independently conducted by two researchers. If there were discrepancies, a third researcher made the final decision. for each outcome into four levels: very low, low, medium, and high (22) pairs.

### Data analysis

We conducted a meta-analysis using Review Manager 5.4 and Stata 17.0 software. The outcomes of this study were continuous variables. Given the diverse origins of heterogeneity and the employment of various measurement scales, the effect size for this research was determined using the standardized mean difference (SMD) and 95% confidence intervals (CI). SMD values of 0.2, 0.5, and 0.8 indicate small, medium, and large significant effects, respectively (22). In this study, we analyzed mental health outcomes such as anxiety and depression, as well as fatigue, sleep, and quality of life. We used the Cochran’s Q statistic to measure the extent of statistical heterogeneity in this study. The choice of the effect model depends on four factors: the goal of statistical inference, the number of studies, the magnitude of statistical heterogeneity, and the presence of a common effect (23). We followed the selection flowchart provided by Tufanaru et al. (23). to choose the appropriate effect model. According to the recommendations of the Cochrane Handbook, when there are sufficient studies (usually ≥10), a random-effects model should be used to address potential heterogeneity (22). In this study, our goal was to make general inductive conclusions. Therefore, when the number of studies is ≥10, we use a random-effects model for analysis regardless of the level of heterogeneity. When the number of studies is ≤5, our goal is simply to present the results of the included studies, so we use a fixed-effects model. If the number of included studies is ≤5 and heterogeneity is too high (I² > 75%), we do not conduct a meta-analysis but instead use descriptive analysis. When the data cannot be pooled, we also adopt descriptive analysis. For outcome indicators with at least ten published studies, funnel plots and Egger’s test were used to detect publication bias. A p-value < 0.05 was considered to indicate statistical significance. We conducted a subgroup analysis for results with more than 10 studies, based on different guidance methods, intervention durations, number of intervention modules, and control conditions.

### Sensitivity analysis

We conducted a sensitivity analysis using RevMan 5.4 software, switching between the random-effects model and the fixed-effects model for analysis, and carried out the process by sequentially excluding the included studies.

## Results

### Literature search

**Figure 1:**
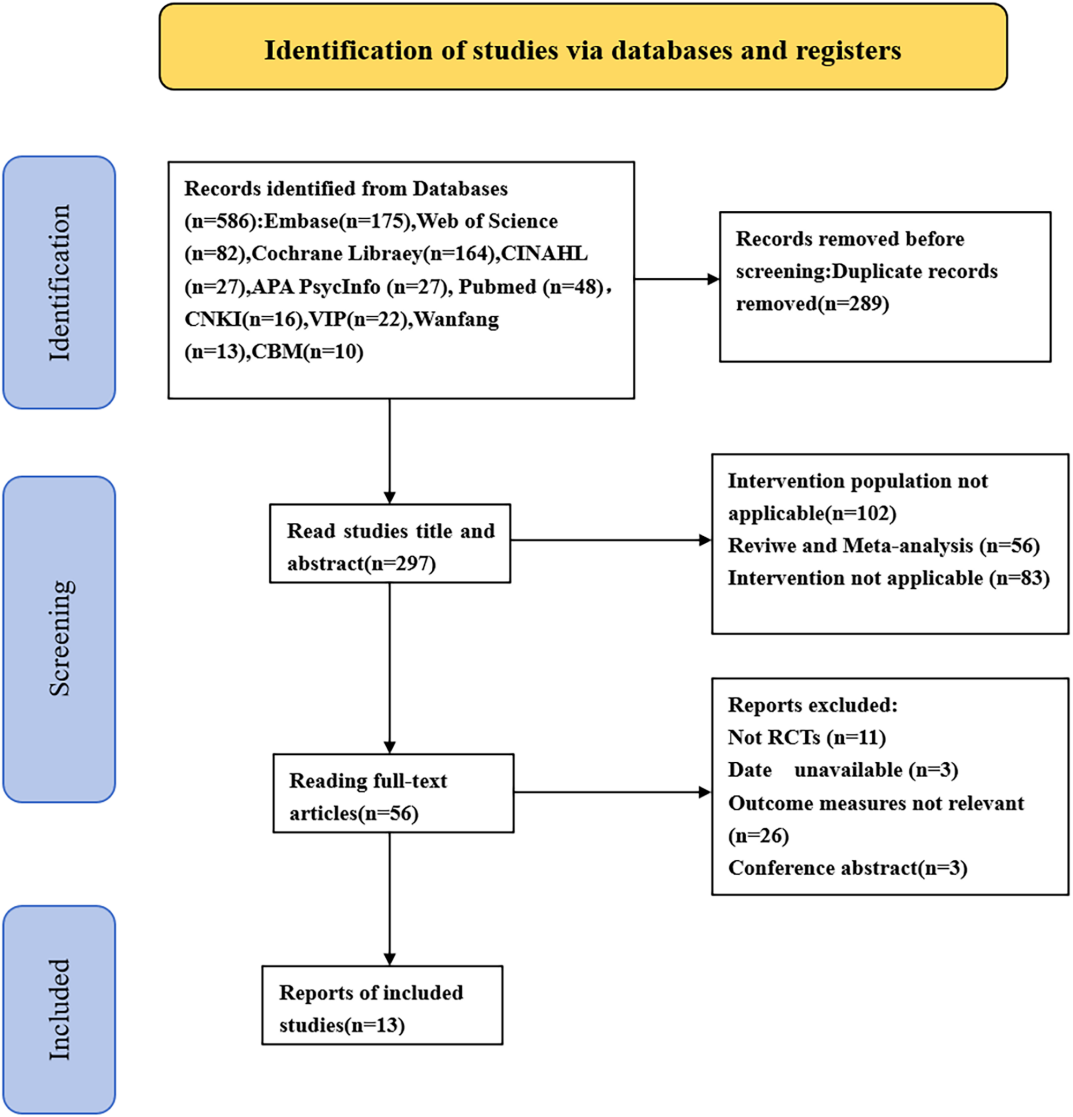
Literature screening flowchart.

In the preliminary search, 586 articles were identified. We excluded 289 duplicate studies, 102 studies where the intervention population did not meet the PICOS criteria, 56 reviews or meta-analyses, and 83 studies where the intervention conditions did not meet the PICOS criteria. Additionally, 11 non-RCT studies, 3 studies with inaccessible full text, 26 studies with irrelevant results, and 3 conference abstracts were excluded. In the end, 13 articles were included. The literature screening process is depicted in **Figure 1**.

### Characteristics of the included studies

A total of 13 studies were included (24–36). One study was from China, one study was from Ireland, one study was from Germany, two studies were from America, four studies were from the Netherlands, and four studies were from Denmark and were published between 2015 and 2023.These studies included 2,079 breast cancer patients, with ages ranging from 47 to 60 years. Of these, 1,171 patients underwent ICBT training. The intervention conditions were all internet-based CBT training, with the number and content of modules designed based on traditional CBT ranging from 3 to 16 modules. Six studies involved therapist-guided ICBT (23, 27–29, 31, 32), six studies involved self-guided ICBT (25, 26, 31, 34–36), and one study included both therapist-guided and self-guided groups (27). Control conditions often involve conventional care, traditional CBT, and waiting for treatment, where the waitlist group refers to providing the same intervention to the control group after the study concluded. Details on the basic characteristics of the subjects, intervention content, and more can be found in **Table 1**. We found no clear correlation between the intervention duration and the number of modules. For example, studies with fewer modules had longer intervention durations.

**Table 1:** Characteristics of included studies.

Study	Country	Participants	Age (mean ± SD)	Intervention group	Control group	Number of intervention modules	Intervention frequency and duration	Indicator	Outcome measures assessment tools
Abrahams 2017 (24) Akkol 2023 (25) Amidi 2022 (26) Atema 2019 (27) Damholdt 2016 (28) Ferguson 2016 (29) Gong 2021 (30) Holtdirk 2021 (31) Hummel 2017 (32) Nissen 2019 (33) Van 2015 (34) Wagner 2021 (35) Zachariae 2018 (36)	Netherlands Ireland Denmark Netherlands Denmark American China Gemany Netherlands Denmark Netherlands America Denmark	T:61 C:64 T:49 C:23 T:77 C:54 T1:85 T2:85 C:84 T:94 C:63 T:21 C:14 T:37 C:37 T:181 C:182 T:84 C:85 T:104 C:46 T:70 C:80 T:97 C:99 T:103 C:100	T:52.5 ± 8.2 C:50.5 ± 7.6 T:47.12 ± 7.92 C:49.30 ± 9.66 T:53.5 ± 8.9 C:54.0 ± 7.8 T1:47.5 ± 5.14 T2:47.7 ± 5.73 C:47.0 ± 5.50 T:54.98 ± 8.51 C:54.56 ± 8.74 T:54.00 ± 12.82 C:55.61 ± 11.39 T:45.24 ± 5.04 C:47.03 ± 5.84 T:50.07 ± 8.51 C:49.8 ± 7.98 T:51.60 ± 7.70 C:50.50 ± 6.80 UNCLEAR T:51.44 ± 8.30 C:50.18 ± 9.15 T:54.1 ± 10.1 C:55.3 ± 9.4 T:53.2 ± 8.8 C:52.9 ± 8.9	ICBT(face-to-face and electronic consultations):Interventions are tailored to the patient: 1)set their treatment goals; 2)poor coping with breast cancer;3) high fear of cancer recurrence; 4) dysfunctional fatigue-related cognitions; 5) a deregulated sleep-wake rhythm; 6) a deregulated activity pattern; and/or 7) negative social interactions and low social support; 8) participants realized their treatment goals. ICBT(self-guided):1)quizzes; 2)goal setting; 3)mood monitoring; 4)activity scheduling; 5)thoughts‐feelings‐behaviors cycle; 6)worry tree; 7)relaxation exercises ICBT(self-guided):1)treatment rationale; 2)sleep restriction; 3)stimulus control; 4)cognitive restructuring; 5)sleep hygiene; 6)relapse prevention ICBT(T1:therapist-guided:telephone and email-based support,T2:self-guided):1)welcome; 2)hot flushes; 3)from stressing to relaxing;4)improving sleep; 5)my body and sexuality; 6)keep progressing ICBT(therapist-guided):1)attention; 2)processing speed; 3)learning; 4)memory; 5)working memory; 5)problem-solving ICBT(therapist-guided:videoconference):1)education concerning chemotherapy- related cognitive dysfunction; 2)self-awareness training; 3)stress management and self-regulation; 4)cognitive compensatory strategies training ICBT(therapist-guided): 1) cognitive therapy (animated video); 2) cognitive consolidation (game form: Answer questions + video); 3) celaxation therapy (audio + immersion Style relaxation) ICBT(self-guided):four content domains: 1)psychological well-being; 2) dietary coaching; 3) physical activity and exercise; 4) sleep management. ICBT(therapist-guided:telephone and email-based support): Each module contains several interventions, each of which comprises the following elements: 1) introduction; 2) psychoeducation; 3)”homework” assignments; 4) reporting back to the therapist and receiving feedback on the homework assignments ICBT(therapist-guided): 1)written material; 2) audio exercises; 3) writing tasks; 4) cancer‐specific patient examples; 5) videoswith patients and experts ICBT(self-guided): Four phases of adjustment to breast cancer: 1) looking back; 2) emotional processing; 2) strengthening; 4) looking ahead) ICBT(self-guided): 1) Relaxation; 2) Cognitive restructuring; 3) Scheduled worry practice ICBT(self-guided): 1)introduction and treatment rationale (Core 1);2) sleep restriction and stimulus control (Core 2 and 3);3)cognitive restructuring (Core 4); 4)sleep hygiene (Core 5);5) relapse prevention (Core 6)	waiting list group usual treatment waiting list group waiting list group waiting list group behavioral placebo usual treatment waiting list group waiting list group waiting list group usual treatment CBT waiting list group	8 7 6 6 6 4 3 16 10 8 16 3 6	uc,24weeks uc,8weeks uc,9weeks once a week, 6 weeks 5 days/week, 6 weeks once a week, 8 week uc once a week, 12 weeks once a week, 20 weeks uc,10weeks Once a week,16weeks uc,4weeks uc,9weeks	(5) (6) (1) (2) (6) (3) (4) (5) (1) (2) (4). (1) (2) (1) (2) (6) (1) (2) (3) (1) (2) (3) (5) (6) (1) (2) (1) (2) (3) (1) (2) (6) (1) (2) (5) (6) (3) (4) (5)	CIS-Fatigue Severity EORTC-QLQ-C30 HADS QOL ISI 、PSQI、FACIT-F HADS、GSQS SCL-90; BDI DASS-21 FACT-Cog impact on QOL SAI、PHQ⁃9、AIS GAD-7. PHQ-9、ISI、BFI-9 WHOQOL-BREF HADS STAI‐Y、BDI‐II、ISI HADS、CIS PROMIS Computer Adaptive Tests Sleep Disturbances T-score PROMIS Global Health–T-score ISI、PSQI、FACIT-F

(1) anxiety;(2) depression;(3) insomnia;(4) sleep quality;(5) fatigue;(6) quality of life

uc, unclear

CIS-Fatigue Severity, Checklist Individual Strength Fatigue Severity subscale; EORTC-QLQ-C30, European Organization for Research and Treatment of Cancer Quality of Life Questionnaire Core 30; ICBT, Internet-based cognitive behavioral therapy; Mo, months; QOL, quality of life; SIP-8, Sickness Impact Profile 8. HADS, Hospital Anxiety and Depression Scale; iCBT, internet‐delivered cognitive behavioral therapy; MOS‐SSS, Medical Outcomes Study Social Support Survey; QOL, Quality of Life Scale. PSQI: Pittsburg Sleep Quality Index; ISI: Insomnia Severity index; FACIT-F: The Functional Assessment of Chronic Illness Therapy e Fatigue;。GSQS,Groningen Sleep Quality Scale. SCL-90: the symptom checklist 90, subscale SCL-ANX4 (anxiety), BDI: beck depression inventory. DASS-21: depression anxiety stress scale. FACT-Cog Impact on Quality of Life scale.PHQ⁃9, Patient Health Questionnaire⁃9。AIS, Athens Insomnia Scale。, GAD-7, Generalized Anxiety Disorder。BFI-9, Brief Fatigue Inventory。WHOQOL-BREF, world Health Organization Quality of Life Questionnaire 。STAI‐Y, State‐Trait Anxiety Inventory Y‐Form。BDI‐II, Beck Depression Inventory。CIS, Checklist Individual Strength.

### Risk of bias in individual trials

**Figure 2:**
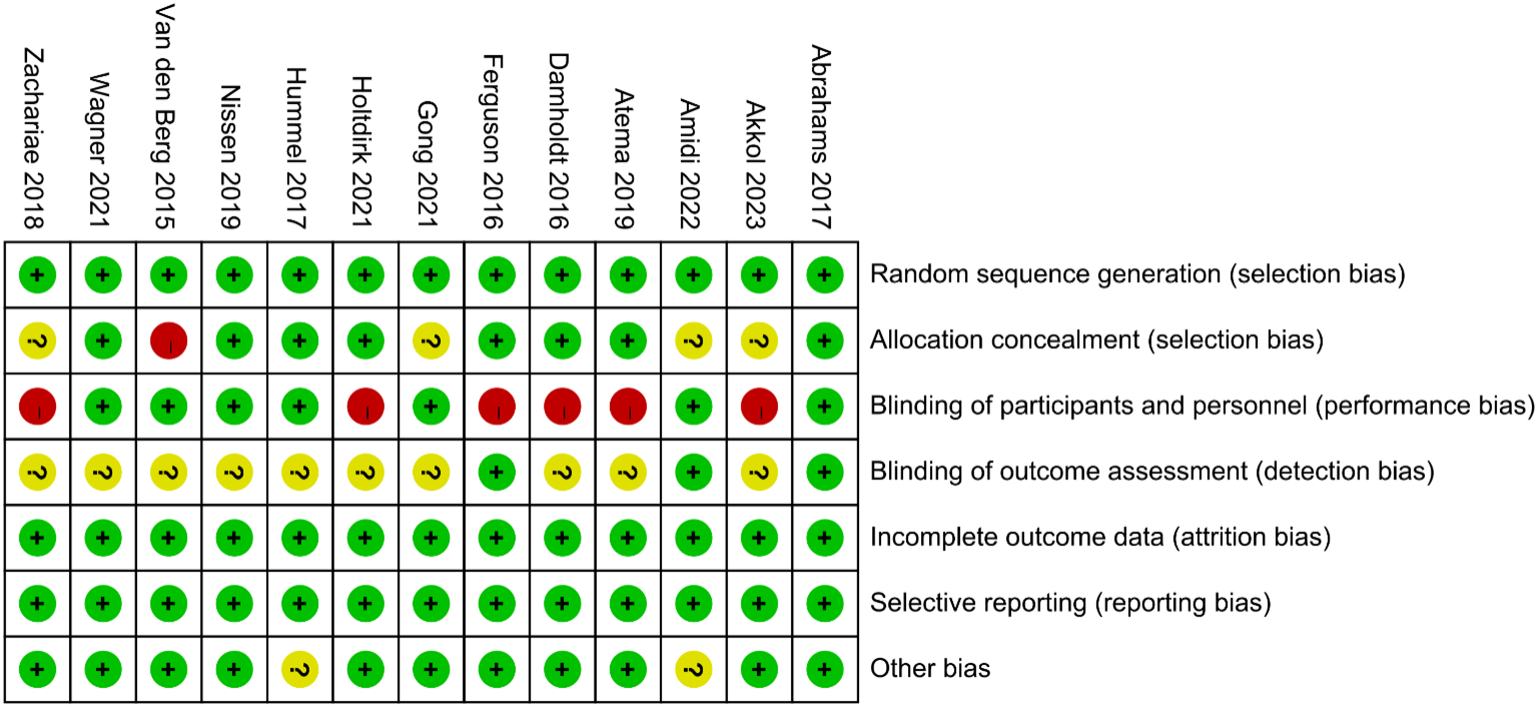
Risk of bias assessment chart.

**Figure 3:**
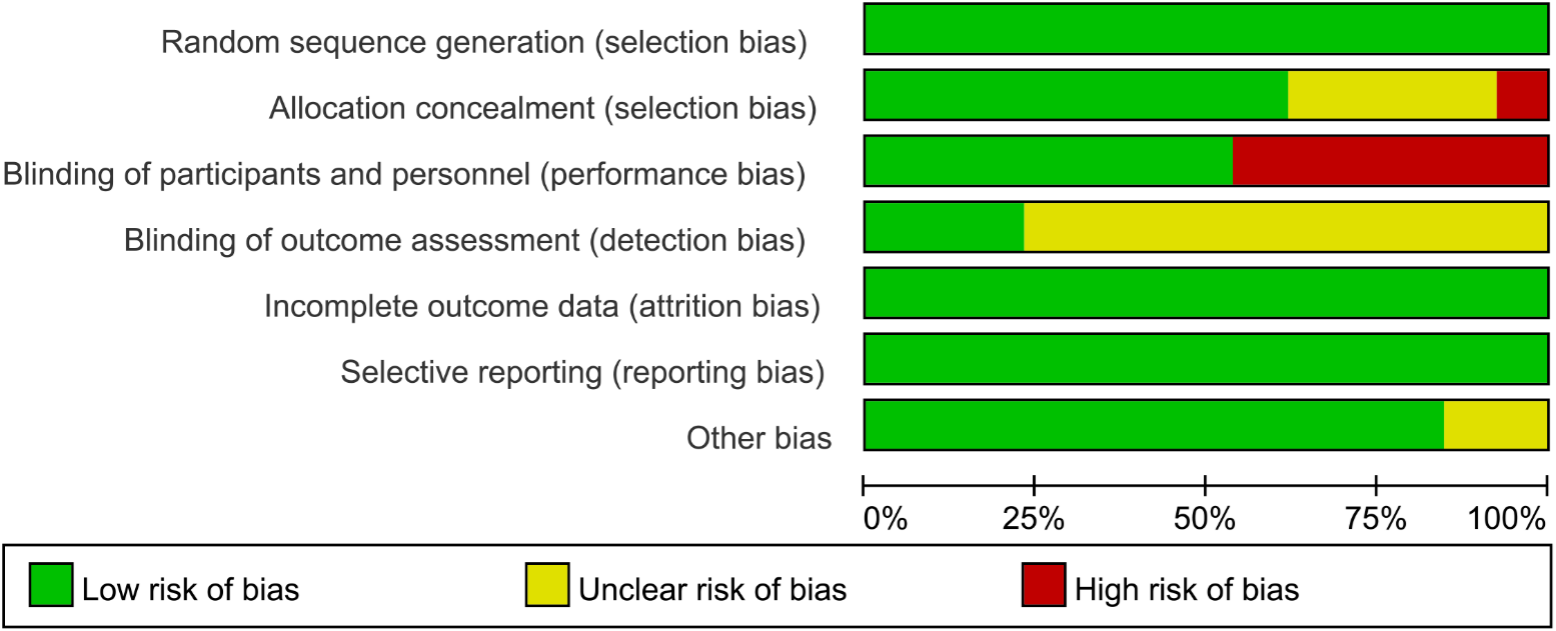
Risk of bias assessment chart (expressed as percentages).

No studies were excluded due to a high risk of bias, as the assessment of bias risk is illustrated in the figure (**Figures 2**, **3**). All included studies described the generation of random sequences, the outcomes were fully reported, and the data were complete; thus, we assessed these three aspects as low risk. One study mentioned that the allocation outcomes were not concealed, so it was assessed as high risk. Four studies did not describe the method of concealment for the allocation scheme; therefore, they are considered to have an unclear risk of bias. Seven studies explicitly reported the use of blinding for participants and personnel and were thus assessed as low risk. Only three studies explicitly mentioned the blinding of outcome assessors, while the rest did not (**Figures 2**, **3**).

### Evidence rating

The overall evidence level ranges from low to moderate. The quality of evidence for anxiety, depression, and sleep quality was moderate, while the evidence for fatigue, insomnia, and quality of life was considered low (**Appendix S2**).

### Results of the meta-analysis

**Figure 4:**
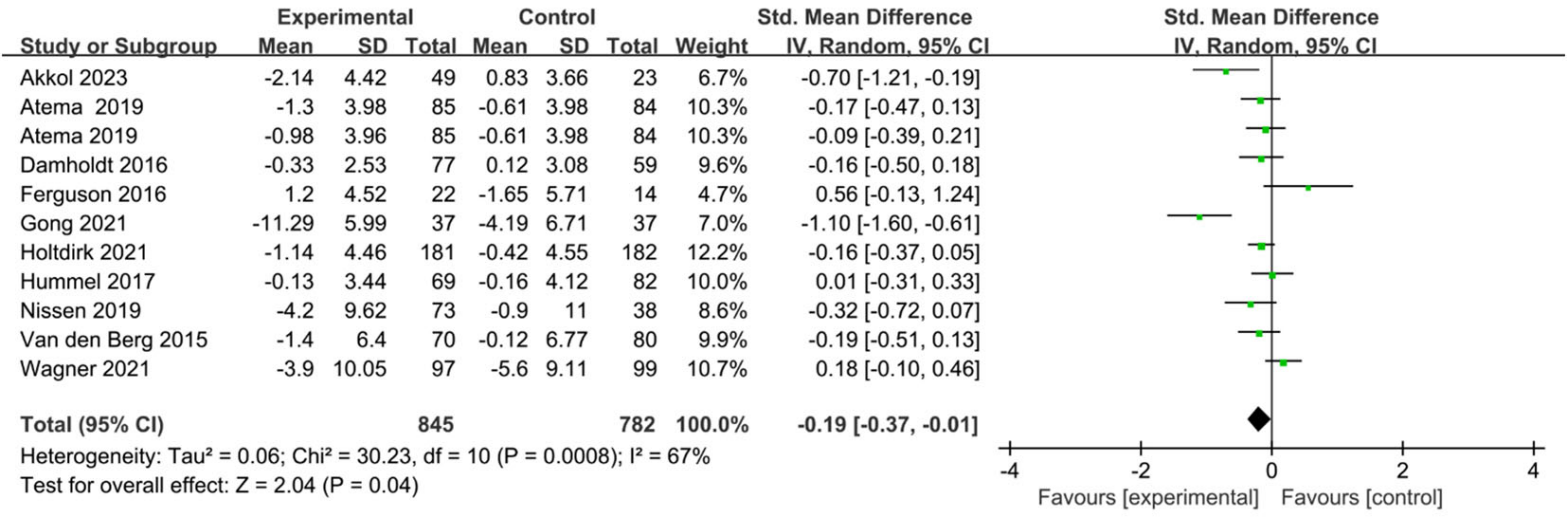
Forest plot of the effect of ICBT on anxiety in breast cancer patients.

**Figure 5:**
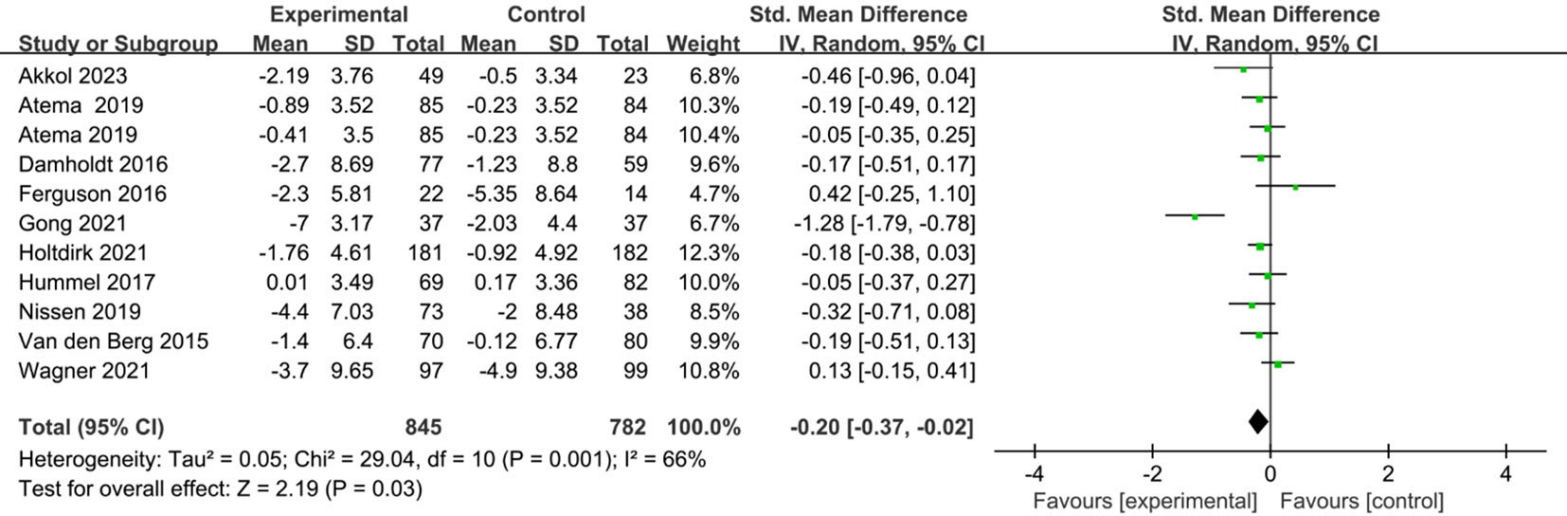
Forest plot of the effect of ICBT on depression in breast cancer patients.

Ten studies comprising eleven comparisons investigated the effects of ICBT on depression and anxiety in breast cancer patients (25, 27–35). We conducted the analysis using a random-effects model. The results showed that the anxiety levels of the breast cancer patients in the experimental group were significantly lower than those in the control group [SMD=-0.19, 95% CI (-0.37, -0.01), P=0.04] (**Figure 4**). The results indicated that the depression levels in the breast cancer patients in the experimental group were significantly lower than those in the control group [SMD=-0.20, 95% CI (-0.37, -0.02), P=0.03] (**Figure 5**).

**Figure 6:**
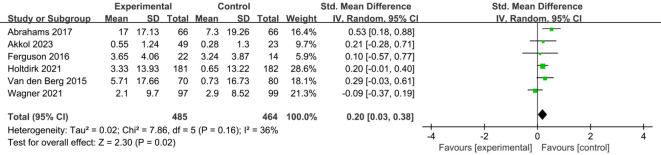
Forest plot of the impact of ICBT on the quality of life of breast cancer patients.

**Figure 7:**
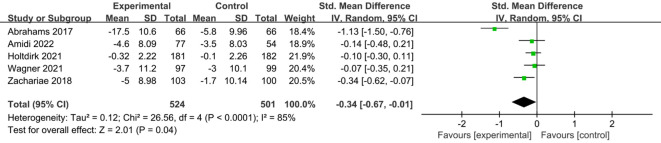
Forest plot of the effect of ICBT on fatigue in breast cancer patients.

**Figure 8:**
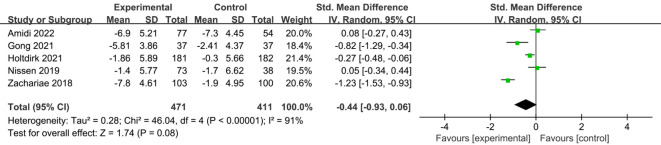
Forest plot of the effect of ICBT on insomnia in breast cancer patients.

**Figure 9:**
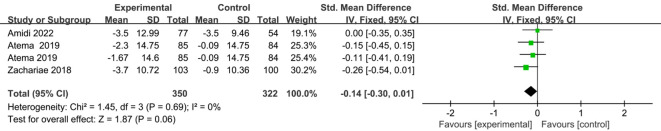
Forest plot of the effect of ICBT on sleep quality in breast cancer patients.

A total of six studies (24, 25, 29, 31, 34, 35) reported on the impact of ICBT on quality of life in breast cancer patients. Although there was low heterogeneity among the included studies (I² = 36%, P = 0.016), we opted for a random-effects model for the analysis and the results showed that the quality of life of patients in the experimental group was significantly greater than that of patients in the control group [SMD=0.20, 95% CI (0.03, 0.38), P=0.02] (**Figure 6**). Five studies (26, 30, 31, 33, 35) reported on the impact of ICBT on fatigue levels in breast cancer patients. Meta-analysis was performed using a random-effects model, and the results indicated that ICBT could reduce fatigue levels in breast cancer patients [SMD = -0.34, 95% CI (-0.67, -0.01), P = 0.04] (**Figure 7**). Five studies (26, 30, 31, 33, 35) reported on the effect of ICBT on insomnia in breast cancer patients, We chose a random-effects model for the analysis and the results indicated that the incidence of insomnia in the experimental group was lower than that in the control group, but the results were not statistically significant [SMD=-0.44, 95% CI (-0.93, 0.06), P=0.08] (**Figure 8**). Three studies (26, 27, 36) reported on the impact of ICBT on sleep quality in breast cancer patients, with one study including two control groups, thus analyzing four sets of data in total. No heterogeneity was observed among the included studies [I²=0%, P=0.69], and the number of studies was less than five; therefore, a fixed-effects model was used for the meta-analysis, indicating that ICBT had no significant effect on improving sleep quality in breast cancer patients [SMD=-0.14, 95% CI (-0.30, 0.01), P=0.06] (**Figure 9**).

### Subgroup analysis

We conducted subgroup analyses on anxiety and depression, based on the mode of guidance (self-guided, therapist-guided), intervention duration (≤8 weeks, >8 weeks), number of modules (≤6, >6), and differences in control conditions.

The subgroup analysis results for anxiety were as follows: regardless of whether therapist-guided ICBT [SMD = -0.22, 95% CI (-0.54, 0.10), P = 0.18] or self-guided ICBT [SMD = -0.14, 95% CI (-0.35, 0.07), P = 0.19] was used, there was no statistically significant difference. In other words, the intervention effect of ICBT on anxiety in breast cancer patients was not associated with the mode of guidance. Compared to an intervention duration ≤8 weeks [SMD = -0.09, 95% CI (-0.33, 0.15), P = 0.49], an intervention duration >8 weeks [SMD = -0.15, 95% CI (-0.30, -0.01), P = 0.03] had a better and more statistically significant effect. The intervention period ≤12 weeks [SMD = -0.12, 95% CI (-0.30, 0.05), P = 0.15] showed no statistical significance, indicating that the intervention effect was not significant. Similarly, for an intervention period >12 weeks [SMD = -0.09, 95% CI (-0.32, 0.13), P = 0.43], the results were also not significant. When the number of intervention modules in the ICBT was >6 [SMD = -0.21, 95% CI (-0.39, -0.04), P = 0.02], the effect was greater than when the number of modules was ≤6 [SMD = -0.14, 95% CI (-0.47, 0.18), P = 0.38]. Finally, concerning control conditions, the results for the waitlist control group were statistically significant [SMD = -0.14, 95% CI (-0.26, -0.02), P = 0.02], while those for the usual care group were not statistically significant [SMD = -0.42, 95% CI (-0.96, 0.11), P = 0.12] (**Table 2**).

**Table 2:** Results of the anxiety subgroup analysis.

Outcome type	Number of studies	*I²*	*P*	Effect model	Meta-analysis results		
					SMD(95%CI)	Z	P
Intervention format							
Therapist-guided Self-guided	6 5	75% 59%	0.05 0.001	Random Random	-0.22[-0.54,0.100] -0.14[-0.35,0.07]	1.35 1.32	0.18 0.19
Duration							
≤8 wk >8 wk ≤12 wk >12 wk	6 4 8 2	61% 0% 51% 0%	0.03 0.62 0.04 0.39	Random Fixed Random Fixed	-0.09[-0.33,0.15] -0.15[-0.30,-0.01] -0.12[-0.30,0.05] -0.09[-0.32,0.13]	0.70 2.13 1.42 0.80	0.49 0.03 0.15 0.43
Number of modules							
≤6 >6	5 5	79% 32%	0.0002 0.21	Random Random	-0.14[-0.47,0.18] -0.21[-0.39,-0.04]	0.88 2.36	0.38 0.02
Control conditions							
waiting list group usual treatment placebo	6 4 1	0% 87%	0.387 <0.0001	Random Random	-0.14[-0.26,-0.02] -0.42[-0.96,0.11] 0.56[-0.13,1.24]	2.35 1.55 1.59	0.02 0.12 0.02

SMD, standardized mean difference effect size.

The subgroup analysis results for depression revealed that neither therapist-guided effects [SMD = -0.27, 95% CI (-0.31, -0.03), P = 0.12] nor self-guided effects [SMD = -0.11, 95% CI (-0.24, 0.02), P = 0.09] were statistically significant. In other words, the intervention effect of ICBT on depression in breast cancer patients was not associated with the mode of guidance. The intervention effect for those with an intervention duration >8 weeks [SMD = -0.17, 95% CI (-0.96, 0.11), P = 0.12] was better than that for those with an intervention duration ≤8 weeks [SMD = -0.07, 95% CI (-0.21, 0.07), P = 0.35]. For an intervention period ≤12 weeks [SMD = -0.12, 95% CI (-0.23, -0.01), P = 0.04], the result was statistically significant, indicating a significant intervention effect. However, for an intervention period >12 weeks [SMD = -0.12, 95% CI (-0.35, 0.11), P = 0.30], the result was not significant. The effect of the number of intervention modules >6 [SMD = -0.19, 95% CI (-0.33, -0.06), P = 0.006] was greater than that of ≤6 [SMD = -0.19, 95% CI (-0.53, 0.15), P = 0.28]. The effect in the waitlist control group [SMD = -0.15, 95% CI (-0.27, -0.03), P = 0.01] was greater than that in the usual care group [SMD = -0.42, 95% CI (-0.96, 0.12), P = 0.13] (**Table 3**).

**Table 3:** Results of the depression subgroup analysis.

Outcome type	Number of studies	*I²*	*P*	Effect model	Meta-analysis results		
					SMD(95%CI)	Z	P
Intervention format							
Therapist-guided Self-guided	6 5	77% 26%	0.0005 0.25	Random Fixed	-0.27[-0.61,0.07] -0.11[-0.24,0.02]	1.57 1.71	0.12 0.09
Duration							
≤8 wk >8 wk ≤12 wk >12 wk	6 4 8 2	30% 0% 21% 0%	0.21 0.71 0.26 0.53	Fixed Fixed Fixed Fixed	-0.07[-0.21,0.07] -0.17[-0.31,-0.03] -0.12 [-0.23,-0.01] -0.12[-0.35,0.11]	0.94 2.37 2.10 1.03	0.35 0.02 0.04 0.30
Number of modules							
≤6 >6	6 5	81% 0%	<0.0001 0.68	Random Fixed	-0.19[-0.53,0.15] -0.19[-0.33,-0.06]	1.08 2.77	0.28 0.0006
Control conditions							
waiting list group usual treatment placebo	6 4 1	0% 87%	0.90 <0.0001	Random Random	-0.15[-0.27,-0.03] -0.42[-0.96,0.12] 0.42[-0.25,1.10]	2.49 1.53 1.23	0.01 0.13 0.22

SMD, standardized mean difference effect size.

### Sensitivity analysis

For all outcomes except fatigue, switching effect models and excluding individual studies had minimal impact on the results, indicating that the findings are robust. However, for the fatigue outcome, excluding the study by Zachariae had a significant impact on the results (36), suggesting that this part of the statistical analysis is less stable.

### Publication bias

**Figure 10:**
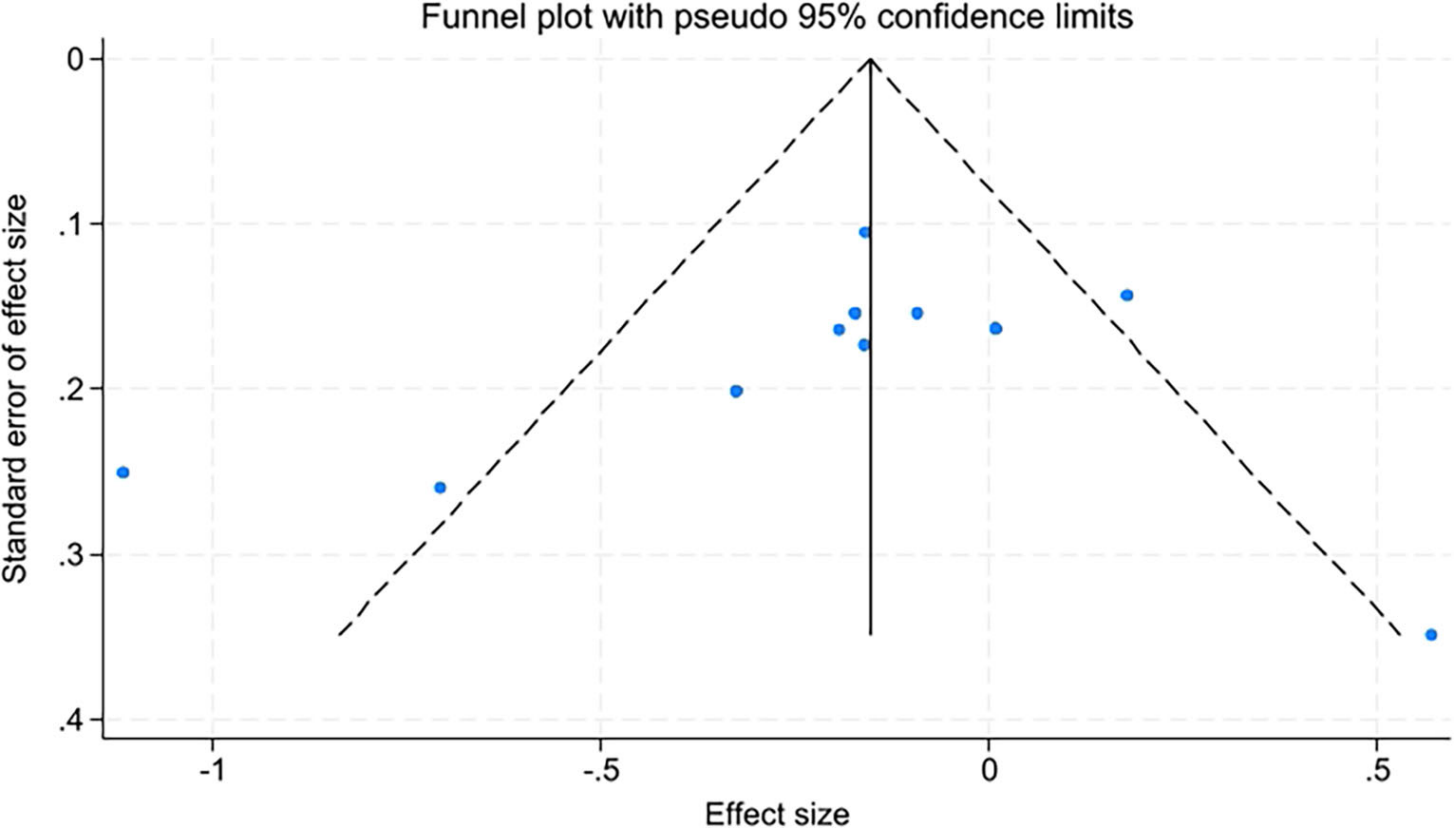
Funnel plot of anxiety.

**Figure 11:**
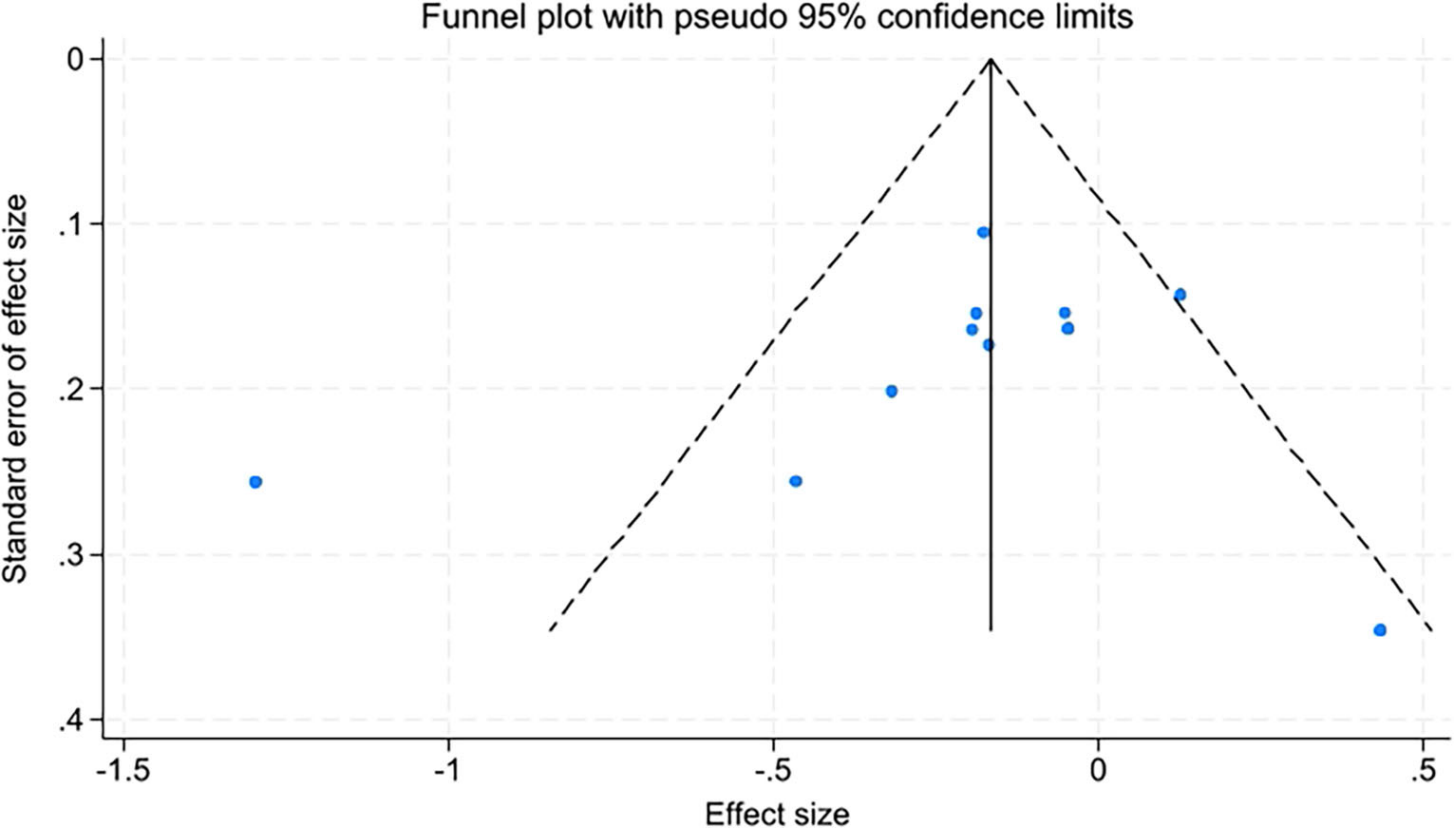
Funnel plot of depression.

Due to the number of studies on quality of life, fatigue levels, insomnia, and sleep quality being fewer than ten, only anxiety and depression were assessed for publication bias using funnel plots and Egger’s test. The funnel plots appeared relatively symmetrical, with Egger’s test results for anxiety at t=-0.89, P=0.398, and for depression at t=-0.92, P=0.381. P-values greater than 0.05 indicate no significant publication bias (**Figures 10**, **11**).

## Discussion

This is the first systematic review and meta-analysis to explore the effects of ICBT on negative emotions and quality of life in breast cancer patients. The results of this study indicate that ICBT can alleviate negative emotions in breast cancer patients. These results align with the meta-analysis by Liu et al. (21), who evaluated the impact of ICBT on anxiety and depression in cancer patients, and reported that ICBT can alleviate negative emotions in such patients, with five of these articles were also included in the present study (27–29, 31, 32).

Our study found that ICBT can alleviate anxiety and depression in breast cancer patients, which is consistent with the meta-analysis results of Liu and Yu et al. (17, 18). ICBT is based on CBT and helps patients regain normal cognitive patterns through cognitive training, equipping them with methods to combat negative emotions and thereby alleviating such emotions. Through online learning, patients can learn in an intuitive way, such as using animations, and provide timely feedback on issues they encounter, helping them experience their sense of self-worth during the learning and training process. Breast cancer patients often suffer from sleep disorders, with up to 80% of them experiencing such issues (34). Moreover, research shows that insomnia can promote the progression of cancer and increase mortality (35). The American Academy of Sleep Medicine recommends using Cognitive Behavioral Therapy for Insomnia (CBTI) to treat chronic primary insomnia. We conducted a meta-analysis on the severity of insomnia and sleep quality in breast cancer patients. The results showed that ICBT had no statistically significant effect on the severity of insomnia or sleep quality in breast cancer patients. In other words, ICBT did not reduce insomnia severity nor improve sleep quality in these patients. Upon analyzing the differences between ICBT and CBTI, we found that ICBT typically includes only one module for sleep training, whereas CBTI focuses more intensively on sleep regulation training. This difference may explain the divergent results. However, due to the limited number of related studies, we did not conduct a subgroup analysis or assess publication bias, so the results should be interpreted with caution. Additionally, psychological distress is also a major cause of poor sleep quality in breast cancer patients (36), and further research is needed to find more effective methods to improve their sleep quality. Up to 99% of patients suffer from cancer-related fatigue (37). A network meta-analysis by Yuan et al. showed that CBT can significantly improve cancer-related fatigue symptoms (38). To explore whether ICBT can alleviate fatigue in breast cancer patients, we also conducted a meta-analysis. The results indicated that ICBT can significantly reduce fatigue levels in breast cancer patients. However, due to the limited number of fatigue-related studies, we did not perform a subgroup analysis or assess publication bias. The impact of ICBT on fatigue in patients needs further research to be confirmed. The study by Yu et al. showed that ICBT did not improve the quality of life of cancer patients (18), whereas our study demonstrated that ICBT can improve the quality of life of breast cancer patients. Given the limited number of included studies and the lack of sensitivity analysis, our results are at risk of bias. Further research specifically targeting breast cancer patients is needed to verify the effectiveness of ICBT in improving their quality of life.

In our study, due to the limited research on insomnia severity, sleep quality, fatigue, and quality of life, we did not conduct subgroup analyses for these factors and only performed subgroup analyses on anxiety and depression. Previous research (17) found that therapist-guided ICBT can improve patient compliance, approximately three times higher than self-guided methods (39). Yu et al. also found that therapist-guided ICBT was more effective. In one of the studies included in our analysis, both self-guided ICBT and therapist-guided ICBT were examined (24). We extracted the data from these two parts separately and compared them. Our results showed no statistically significant difference between therapist-guided and self-guided ICBT, indicating that the effect of ICBT on anxiety and depression in breast cancer patients is independent of the guidance method. The discrepancy between our findings and previous studies may be due to the fact that prior studies included all cancer patients, while we specifically focused on breast cancer patients. We believe that more high-quality studies are needed to compare the advantages, disadvantages, and effectiveness of these two guidance methods. Therefore, these findings should be interpreted with caution. The intervention duration is often related to its effectiveness, as studies in some diseases have shown that the effects of interventions tend to increase over time. However, several studies on ICBT have demonstrated that shorter intervention durations often lead to more effective results (40–42). Our subgroup analysis based on different intervention durations revealed that when the intervention period exceeded 8 weeks, the intervention effect was significant. However, for interventions lasting 8 weeks or less, the effect was not significant. A longer intervention period may cause patients to lose patience, leading to decreased compliance (43), which in turn reduces the effectiveness of the treatment. On the other hand, a shorter intervention period may also result in insignificant effects. Liu et al. conducted a subgroup analysis based on whether the intervention duration exceeded 12 weeks and found that ICBT was more effective for cancer patients when the intervention duration was ≤12 weeks. Although their meta-analysis included all cancer patients, it also provides some reference value for breast cancer patients. Some studies suggest that the long-term intervention effect of ICBT is not ideal (44). Therefore, we conducted a subgroup analysis based on a 12-week cutoff and found that, for depression, the results were not significant regardless of whether the intervention period exceeded 12 weeks. For anxiety, the intervention effect was significant when the duration was ≤12 weeks. Hence, we recommend an intervention period of 8 to 12 weeks. Current ICBT studies typically divide the intervention into different modules, with each module gradually helping patients understand relevant knowledge and training content. We found no correlation between the number of modules and the intervention duration. Studies with longer intervention durations may include fewer modules. Therefore, we also performed a subgroup analysis based on the number of modules. Our study included research with a total of 3 to 16 modules, with most studies containing 6 to 8 modules. We used 6 modules as a cutoff for the subgroup analysis and found that when the number of modules exceeded 6, the intervention effect of ICBT on anxiety and depression in breast cancer patients was better and more significant. A possible reason is that the more modules there are, the richer the content becomes, and the shorter the average duration of each module, which helps maintain patient interest by avoiding long viewing times. Liu et al.’s results showed that when the number of modules was ≥5, the results were more significant. We recommend that for breast cancer patients, having more than 6 modules is preferable. Differences in control conditions can affect the results (45). We conducted a subgroup analysis based on different control conditions and found that the waitlist control group showed significant intervention effects, while the usual care group did not show significant effects, though the estimated SMD was higher than that of the waitlist group. This differs from Yu et al.’s findings (18), which showed that ICBT was more effective in the usual care group than in the waitlist control group. The reason might be that the waitlist control could induce a placebo effect, influencing the results and making the intervention effect closer to the control group. Upon analyzing the two groups’ results, we found that the usual care group exhibited high heterogeneity, which could be the reason for the insignificant effect. Additionally, patients in the waitlist group were less likely to seek other treatments and tended to exhibit a more positive attitude during the treatment period. Our study cannot provide a definitive conclusion regarding the superiority of usual care versus waitlist care, so these results should be interpreted cautiously. Further research can delve deeper into this topic.

### Limitations

We systematically searched the databases and identified studies that met the requirements, but there are some unavoidable limitations. The first limitation. Due to the nature of the intervention studies, almost all included studies could not implement blinding for the subjects. The outcome measures were assessed using subjective scales, and the lack of blinding introduces a certain risk of bias. The second limitation is the diversity of outcome assessment tools. The variety of tools used across the included studies prevented us from conducting subgroup analyses based on different assessment tools. The third limitation involves the differing characteristics of the included patients, such as race and cancer staging, which may affect the intervention outcomes. More research is needed to verify these effects in the future. Fourth, the intervention content of ICBT varies across different studies. For example, in studies focusing on sleep, the ICBT intervention content tends to be more oriented toward sleep-related designs, which may influence the results. Furthermore, since it is impossible to determine when the withdrawn patients exited the study, nor the extent of the intervention they received, we are unable to perform a subgroup analysis based on the available information. Therefore, this study did not analyze aspects such as engagement and withdrawal rates. Finally, it was not possible to conduct bias assessments for the effects of ICBT on aspects such as sleep and physical symptoms in breast cancer patients, which may carry a certain risk of bias.

## Conclusions

ICBT can alleviate anxiety and depression in breast cancer patients, with no statistically significant difference between therapist-guided and patient self-guided approaches. The results became statistically significant when the intervention period was longer than 8 weeks and the number of modules exceeded 6. ICBT can reduce fatigue and improve quality of life for breast cancer patients, but it does not significantly improve patients’ sleep. Future research of higher quality is still needed.

## Data Availability

The original contributions presented in the study are included in the article/supplementary material. Further inquiries can be directed to the corresponding author.
